# Fabrication and characterization of a new eco-friendly sulfonamide-chitosan derivative with enhanced antimicrobial and selective cytotoxicity properties

**DOI:** 10.1038/s41598-024-60456-1

**Published:** 2024-05-03

**Authors:** Ahmed G. Ibrahim, Ahmed G. Hamodin, Amr Fouda, Ahmed M. Eid, Walid E. Elgammal

**Affiliations:** 1 Department of Chemistry, Faculty of Science (Boys), Al-Azhar University, Nasr City, El-Nasr Road, Cairo, 11884 Egypt; 2 Department of Botany and Microbiology, Faculty of Science (Boys), Al-Azhar University, Cairo, Egypt

**Keywords:** Chitosan, Sulfonamide-derivative, Antibacterial, Anticancer, Cytotoxicity, Cancer models, Biomedical materials, Organic chemistry, Polymer chemistry, Biopolymers, Polymer characterization

## Abstract

Chitosan (CH) exhibits low antimicrobial activity. This study addresses this issue by modifying the chitosan with a sulfonamide derivative, 3-(4-(N,N-dimethylsulfonyl)phenyl)acrylic acid. The structure of the sulfonamide-chitosan derivative (DMS-CH) was confirmed using Fourier transform infrared spectroscopy and Nuclear magnetic resonance. The results of scanning electron microscopy, thermal gravimetric analysis, and X-ray diffraction indicated that the morphology changed to a porous nature, the thermal stability decreased, and the crystallinity increased in the DMS-CH derivative compared to chitosan, respectively. The degree of substitution was calculated from the elemental analysis data and was found to be moderate (42%). The modified chitosan exhibited enhanced antimicrobial properties at low concentrations, with a minimum inhibitory concentration (MIC) of 50 µg/mL observed for *B. subtilis* and *P. aeruginosa*, and a value of 25 µg/mL for *S. aureus*, *E. coli*, and *C. albicans*. In the case of native chitosan, the MIC values doubled or more, with 50 µg/mL recorded for *E. coli* and *C. albicans* and 100 μg/mL recorded for *B. subtilis*, *S. aureus*, and *P. aeruginosa*. Furthermore, toxicological examinations conducted on MCF-7 (breast adenocarcinoma) cell lines demonstrated that DMS-CH exhibited greater toxicity (IC50 = 225.47 μg/mL) than pure CH, while still maintaining significant safety limits against normal lung fibroblasts (WI-38). Collectively, these results suggest the potential use of the newly modified chitosan in biomedical applications.

## Introduction

Currently, there is significant interest in the development of natural polymers for various biological applications. These polymers are valued for their non-toxicity, biocompatibility, and biodegradability^1–3^, making them environmentally friendly and safe for human use. Among the natural polymers, chitin stands out as one of the most important, owing to the abundance of its sources, including shrimp shells, crabs, fungi, and various marine organisms^4,5^. Chitin is a biopolymer composed of 2-acetamido-2-deoxy-D-glucopyranose^6^. When chitin undergoes deacetylation in a strong alkaline medium, it transforms into chitosan^4^. Chitosan possesses amino groups (located at the C_2_-position) and hydroxyl groups (located at the C_3_ and C_6_-position), which impart hydrophilicity and cationic properties under acidic conditions^7,8^. However, the abundance of amino and hydroxyl groups (two hydroxyl and one amino group per glucopyranose unit) results in intramolecular and intermolecular forces between the chitosan chains^1,9^.

Consequently, chitosan exhibits poor solubility in organic solvents, limiting its potential applications across various fields. Chitosan derivatives have been synthesized through chemical modification to address this limitation, and their effectiveness in numerous applications has been investigated. For instance, Pestov et al*.*^10^ prepared N-(5-methylimidazol-4-ylmethyl) chitosan as a chelating agent for copper and nickel ions. Prichystalová et al*.*^11^ designed chitosan-g-amino-anthracene derivatives and examined their fluorescence spectra and antibacterial activity.

Mohamed et al.^4^ fabricated a benzoimidazolyl-thiadiazole chitosan derivative and evaluated its inhibitory efficacy against selected pathogenic microbes. The derivative was incorporated into a topical gel formula using carbopol 940 and carboxymethyl cellulose, exhibiting a release percentage of 89.9–81.6% after 8 h. Chitosan was modified through maleic grafting for water treatment purposes, targeting specific copper and nickel ions^6^. Ahmed et al.^12^ developed a hydrogel matrix by combining an N-aminophthalimide-based heterocyclic compound with chitosan, and assessed its antimicrobial activity. Cui et al.^13^ prepared chitosan derivatives using carboxylic acid derivatives of pyrrole, thiophene, and furan. They found that the prepared derivatives had inhibitory activity against *Fusarium graminearum*, *Fusarium oxysporum*, *Staphylococcus aureus*, and *Escherichia coli*. The chitosan derivatives obtained by introducing hydroxycinnamic acids into chitosan skeleton displayed good antioxidant activity and antibacterial activity against *Staphylococcus aureus* and foodborne pathogens, as well as good cytocompatibilities to cells^14^. Furthermore, chitosan oligosaccharide derivatives with enhanced *in vitro* antibacterial and antioxidant properties were synthesized by reaction between cinnamyl bromide and the primary amino groups of chitosan^15^. Wei et al.^16^ prepared antioxidants based on N,N-dimethyl chitosan. Due to the presence of an amino group at the C_2_ position, several Schiff base derivatives were prepared using various aldehydes, such as 2-hydroxy-3-methoxy benzaldehyde, 4-hydroxy-3-methoxy benzaldehyde, and 2-hydroxy benzaldehyde^17^, 4-((pyridin-2-ylimino)methyl)benzaldehyde^18^, (2-iminothiophenol methyl)benzaldehyde^19^, salicylaldehyde derivatives^20^, 5-(4-Oxo-2-thioxo-thiazolidin-3-ylimino)-pentanal^21^, among others. The literature shows a strong interest in designing new chitosan derivatives for diverse applications^22,23^.

Sulfonamides possess bioactivity and find extensive use in medications, making them a subject of great interest to synthetic chemistry. Within clinical practice, these medications include antibiotics^24^. The term “sulfonamide” refers to any substance that contains the sulfonamide moiety (SO_2_NH_2_). Sulfonamides have garnered significant attention due to their diverse biological activities across pharmaceutical and agricultural sectors. They are frequently used as antibacterial agents^25^, insulin-releasing^26^, carbonic anhydrase inhibitory^27^, anti-inflammatory^28^, antitumor^29^, antioxidant^30^, anticancer, anti-viral agents^31^, and in Alzheimer’s disease^32^. Researchers have endeavored to combine the advantageous properties of chitosan and sulfonamide into a single product. Dragostin et al*.*^7^ reported the synthesis of chitosan modified with sulfonamide drugs, such as sulfadiazine, sulfamerazine, and sulfizoxazol, aiming to improve the biological effects of chitosan as an antioxidant. Additionally, Kandile et al*.*^33^ employed the sulfonamide 2-chloro-N-(4-sulfonylphenethyl)acetamide to enhance the antimicrobial activity of chitosan by chemical modification.

Continuing this interest and our great passion for developing new antimicrobial chitosan derivatives, we present the synthesis of a new sulfonamide-chitosan derivative (DMS-CH) in this work. We first synthesized and characterized 3-(4-(N,N-dimethylsulfonyl)phenyl)-acrylic acid as the sulfonamide derivative using FT-IR, NMR, and mass spectroscopy techniques. The DMS-CH derivative was then extensively characterized and investigated using FT-IR, 1H-NMR, SEM, TGA, and XRD analyses. Here, we attempted to enhance chitosan’s antimicrobial and cytotoxic properties by modifying it with the prepared sulfonamide derivative. To validate our approach, we conducted an agar diffusion assay against a range of pathogenic microbes, including Gram-positive and Gram-negative bacteria, as well as *Candida albicans*. Furthermore, we performed an MTT (3-(4,5-Dimethylthiazol-2-yl)-2,5-Diphenyltetrazolium Bromide) assay to evaluate the cytotoxic potential of the modified chitosan against breast adenocarcinoma cell lines (MCF-7) and normal lung fibroblasts (WI-38).

## Experimental section

### Substrates and reagents

The natural polysaccharide, chitosan (C_6_H_11_NO_4_)_n_, was gained from Sigma-Aldrich (USA). Other reagents, such as phenyl acrylic acid (PA, PhCH = CHCOOH, 99.0%), dimethyl amine (DMA, (CH_3_)_2_NH, 40.0%), thionyl chloride (SOCl_2_, 99.0%), pyridine (C_5_H_5_N, 99.5%), and triethyl amine (C_6_H_15_N:98.5%), were procured from Darmstadt, Germany, and Loba Chemie, India, respectively. Solvents, including dichloromethane (CH_2_Cl_2_, 99.0%), methanol (CH_3_OH, 99.7%), and acetone (CH_3_COCH_3_, > 99.5%), were purchased from Fisher Chemicals (United States) and El-Nasr companies (Egypt), respectively. Distilled water was prepared in our research lab. All reagents and solvents for synthesis were of analytical grade and used without extra purification.

### Molecular weight of chitosan

In our previous work^34^, we determined the viscosity average molecular weight of chitosan (*M*_*v*_) by the method reported in^35^. The determined *M*_*v*_ was found to be 61 × 10^3^ g/mol.

### Degree of deacetylation of chitosan

In our previous work^34^, the degree of deacetylation (DD) of chitosan was calculated by the equation reported in^36^, which was found to be 70.89%.

### Chemical synthesis

#### The technique for the preparation of (E)-3-(4-(N, N-dimethylsulfonyl)phenyl)acrylic acid [DMSA]

0.01 mol of (E)-3-(4-(chlorosulfonyl)phenyl)acrylic acid, PA-SO_2_Cl, was mechanically stirred with 30 mL of anhydrous dichloromethane at ambient temperature. A catalytic amount of an organic base, such as pyridine (2 mL), was added to the mixture. Through a dropping funnel, 0.01 mol of dimethylamine was added drop by drop for 15 min. The resulting mixture was vigorously stirred for 3 h, and the excess solvent was removed under reduced pressure. The solid was filtered by suction, washed with cold water, and dried in air to obtain the crude derivative, DMSA. The crude derivative was then re-crystallized using a CH_3_OH–CH_2_Cl_2_ mixture. [Yield: 78%; Color: brown; Melting point: 236–238 °C].

#### The technique for the preparation of (E)-3-(4-(N,N-dimethylsulfonyl)phenyl) acryloyl chloride [DMSA-Cl]

In a 100-mL one-necked round-bottom flask equipped with a mechanical stirrer, reflux condenser, and separatory funnel, 0.01 mol of the derivative DMSA and 0.13 mol of thionyl chloride were gradually added from the separatory funnel for about 15 min with robust stirring. The temperature of the resulting mixture was raised until it reached boiling under reflux conditions, and the reaction was allowed to proceed for approximately 2 h. After completion of the reaction, the solvent was drawn off under diminished pressure, producing a crude substance ready for the next step without extra purification.

#### The technique for the preparation of DMS-CH

**Scheme 1:**
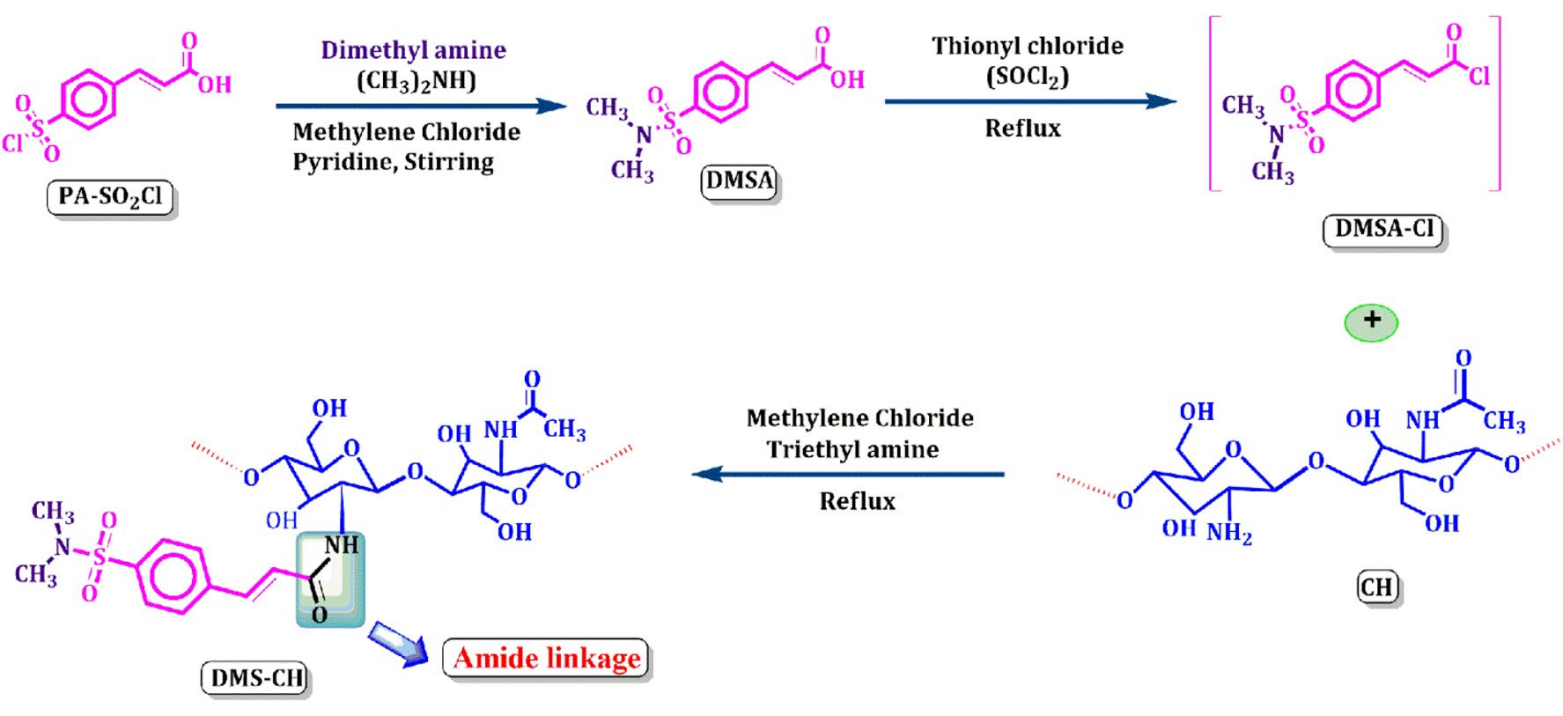
The reaction of chitosan with phenyl acrylic acid derivative linked sulfonamide moiety.

In a round-bottomed flask equipped with a magnetic stirrer and reflux condenser, 0.01 mol of chitosan was dispersed in 30 mL of dichloromethane (DCM). The solution of the crude product of the acid chloride derivative, DMSA-Cl, was added to the flask, followed by the addition of a catalytic amount of triethyl amine (0.02 mol). The materials were heated to boiling and vigorously stirred overnight at 70 °C. The synthesis mixture was poured into excessive acetone when the time ran out. The mixture was filtered and washed multiple times with acetone to purify the newly modified chitosan derivative, as demonstrated in Scheme 1.

### Analytical methods

#### Fourier transform infrared (FT-IR) spectroscopy

Data infrared spectra were recorded on a Shimadzu FT-IR Affinity-1 Spectrometer and a Nicolet iS10FT IR Spectrometer, Thermo Fisher Scientific, ESCALABXi+, USA. The spectra were obtained between 400 and 4000 cm^−1^ with a resolution of 4 cm^−1^ at 25 °C. The sample and potassium bromide (1:100) were mixed, ground into a disc, and scanned in transmittance mode with 16 scans^37^.

#### ^1^H-NMR and ^13^C-NMR spectra

Liquid-state ^1^H/^13^C-NMR spectra were performed using a JNM-ECA 500 II made by JEOL-JAPAN. The spectra were obtained at frequencies of 500 MHz for ^1^H-NMR and 125 MHz for ^13^C-NMR. Chemical shifts are given in parts per million (ppm) relative to an organosilicon compound, tetramethyl silane (TMS, Si(CH_3_)_4_), used as an internal reference in a deuterated solvent, such as dimethyl sulfoxide(CD_3_)_2_S=O). Coupling constants (*J*) are reported in Hertz (Hz). The data are reported as follows: chemical shift, integration, multiplicity (s = singlet, d = doublet, t = triplet, q = quartet, m = multiplet, dd = doublet of doublets, and br = broad), and coupling constants.

#### Mass spectrum and elemental analysis

The acquisition of the mass spectrum was done by the Thermo Scientific Gcms Model (Isq Lt) with Thermo X-Calibur Software (Shimadzu, Kyoto, Japan) at the Regional Center for Mycology and Biotechnology (RCMB), Al-Azhar University, Nasr City, Cairo, Egypt. The measurement of elemental analysis was conducted using the Elemental C-H-N-S Analyzer Vario El M (Germany), and the results were within ± 0.4%.

#### Thermogravimetric analysis (TGA)

The thermal stability of the chitosan and its derivative was determined by the TGA instrument, famous for thermal analysis. This was measured using Discovery SDT 650 simultaneous DSC-TGA/DTG instruments from the USA. Alumina powder was used as a reference material, and the samples were heated from 25 to 700 °C (10 °C/min) under a nitrogen atmosphere with a 30 mL/min flow rate.

#### X-ray diffraction (XRD) analysis

We studied the XRD patterns of chitosan and its derivative using a Siemens D5000 X-ray diffractometer (Germany) equipped with a Cu-Kα radiation source^38^. The samples were scanned over the range of diffraction angle (2θ) from 5° to 40°.

#### Scanning electron microscopy (SEM) analysis

The morphological structure of chitosan and its derivative was examined using a scanning electron microscope (FEI Inspect 5 equipped with an EDX unit, Holland). The accelerating voltage used was 20–30 kV.

### Biological applications

#### Antimicrobial activity studies

The potential antimicrobial properties of pure chitosan, DMSA, and newly adjusted chitosan (DMS-CH) were checked against a selected group of clinical pathogens, covering Gram-negative bacteria (*Pseudomonas aeruginosa* and *Escherichia coli*), Gram-positive bacteria (*Bacillus subtilis* and *Staphylococcus aureus*), and the dimorphic fungus *Candida albicans*. An agar-well diffusion test was performed by using overnight cultures of the tested bacterial strains and *C. albicans*, which were grown in nutrient broth (NB) and yeast extract peptone dextrose (YEPD) broth, respectively. The reproducible culture medium Mueller–Hinton Agar (Oxoid) plates were aseptically poured. To conduct the test, 50 µL of each overnight microbial cultures (concentration = 1.0 O.D.) were evenly swabbed onto Mueller–Hinton plates. Four equidestal holes (0.8 cm) were punched in each inoculated Mueller–Hinton plate^39^. Three stock solutions of CH, DMSA, and DMS-CH were prepared individually by dissolving them in 10% DMSO and serially diluted to the final concentrations of 6.25, 12.5, 25, 50, 100, 200, and 300 µg/mL to evaluate the minimum inhibitory concentration (MIC) for each tested substance. Next, 100 µL of the prepared concentrations of each substance, as well as DMSO as the negative control, were added to the agar wells. The inoculated plates were refrigerated for 1 h and then incubated at 35 ± 2 °C for 24 h. The active compounds were identified by the emergence of visualized clear zones of growth inhibition around the wells. The MIC values were assessed by scaling the diameter (mm) of the developed inhibition zones (ZOI)^40^.

#### In vitro cytotoxicity assessment

The colorimetric MTT assay was employed to investigate the toxicological properties of DMS-CH, DMSA, and CH against the cancer cell lines MCF-7 (breast, mammary gland epithelial adenocarcinoma) and the normal clones WI-38 (lung fibroblast) (provided by VACSERA, Egypt). MCF-7 and WI-38 cells were cultured in RPMI medium supplemented with 2% serum and incubated at 37 °C in a humidified atmosphere (50 mL/L CO_2_) with a cell density of 1×10^5^/100 µL. After one day of incubation, the culture medium was decanted, and the confluent cell layer was washed twice with washing media. Double-fold dilutions (1000˗31.25 μg/mL) of DMS-CH, DMSA, and CH were prepared in RPMI media. Then, the obtained cell sheets were treated with the prepared sample concentrations (100 μL/well). Three wells, including maintenance media (RPMI + 2% serum) only, served as controls, and the treated plates were incubated for 48 h. By the end of incubation, the maintenance media were decanted, and each well received 50 μL of MTT solutions (5 mg/mL, Bio Basic Canada Inc.). The plates were then covered and shaken for 10 min at 150 rpm and re-incubated for 3 h^41^. Finally, the MTT medium was aspirated and replaced with 10% DMSO (200 μl/well) to dissolve the formed formazan crystals. The impact of different treatments on cell viability was evaluated by measuring O.D. at 570 nm using an ELISA microplate reader, and cell viability was determined by the equation^42^:

1 $${\text{Cell}}\;{\text{viability}}\left( {\text{\%}} \right) = \frac{{{\text{Absorbance}}\;{\text{of}}\;{\text{treated}}\;{\text{sample}}}}{{{\text{Absorbance}}\;{\text{of}}\;{\text{control}}}} \times 100$$

## Results and discussion

### Chemistry

As shown in Scheme 1, the DMS-CH derivative is formed through the synthesis of chemical amide linkages between phenyl acrylic acid sulfonamide and chitosan (CH). The derivative (DMSA) was achieved through the reaction of (E)-3-(4-(chlorosulfonyl)phenyl)acrylic acid (PA-SO_2_Cl) with dimethyl amine (aliphatic amine) under stirring conditions with a catalytic amount of pyridine^43,44^. The chlorination reaction was then conducted using thionyl chloride as the chlorinating agent, resulting in the formation of a non-isolable intermediate acid chloride derivative (DMSA-Cl), which was then coupled with the primary amino groups of the chitosan macromolecule (CH) to give the desired functionalized chitosan (DMS-CH), as depicted in Scheme 1.

### Characterization

#### FT-IR studies

**Figure 1:**
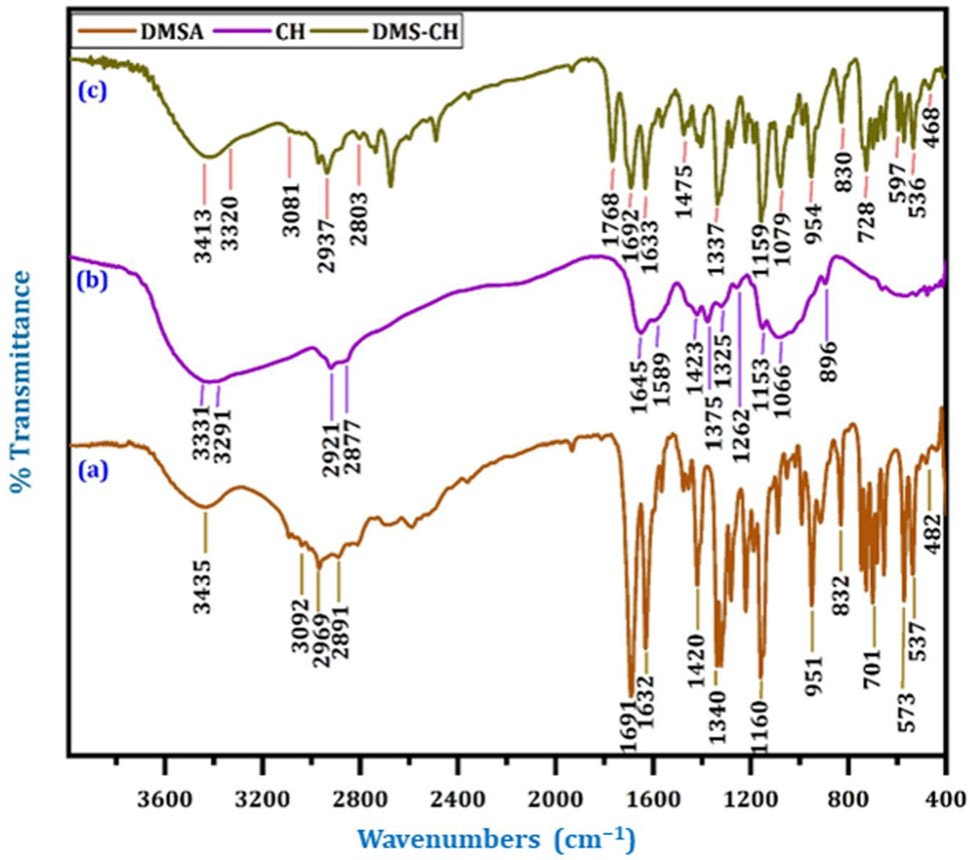
The FT-IR spectra of (**a**) DMSA, (**b**) CH, and (**c**) DMS-CH.

The FT-IR data furnished the chemical structures of DMSA, CH, and DMS-CH (Fig. 1a–c). In the FT-IR spectrum for DMSA (Fig. 1a), the band at 3435 cm^−1^ was assigned to the hydroxyl group (O–H) stretching absorption, while the bands at 3092 cm^−1^, 2969 cm^−1^ and 2891 cm^−1^ were ascribed to aromatic and aliphatic (C–H) stretching absorption, respectively. The absorption band related to the carbonyl (C=O) of the carboxylic group appeared at 1691 cm^−1^, while the absorption band at 1632 cm^−1^ was assigned to the (C=C) group. The symmetric and asymmetric bands of the sulfonamide group (O=S=O) appeared at 1340 cm^−1^ and 1160 cm^−1^. On the other hand, the FT-IR spectrum of original chitosan CH (Fig. 1b) presents a broad band at 3331 cm^−1^ and 3291 cm^−1^, which are attributed to the stretching vibrations of O–H and N–H, respectively. The absorption bands at 2921 cm^−1^ and 2877 cm^−1^ corresponded to the symmetric and asymmetric aliphatic group (–NHCO–CH_3_). The absorption bands at 1645 cm^−1^, 1589 cm^−1^, 1423 cm^−1^, and 1373 cm^−1^ were associated with the existence of the (C=O, amide I) group, bending vibrations related to the N–H (N-acetylamino glucose mers) and amide II, C-N bending (amino group axial deformation (amide III)), and C–N of amide group axial deformation), respectively. The characteristic absorption bands at 1153 cm^−1^, 1066 cm^−1^, and 898 cm^−1^, corresponded to –COC– of (β-(1,4)-glycosidic bonds between chitosan mers), C–O stretching of primer hydroxyl groups (CH_2_–OH), and glucopyranose rings, respectively. In the FT-IR spectrum of DMS-CH (Fig. 1c), the characteristic bands of both DMSA and CH were observed, indicating the successful synthesis of the DMS-CH derivative. The spectrum of DMS-CH exhibits absorption bands ranging from 3413 to 3320 cm^−1^, which can be caused by the presence of the hydroxyl group and the secondary amine. Additionally, an absorption band at 3081 cm^−1^, not visible in the spectrum of chitosan, provides evidence that the modified polymer contains a benzene ring. We also observe a shift of the associated aliphatic absorption bands (C–H). Furthermore, there are three additional bands in the spectrum of the modified polymer that were seen at 1768 cm^−1^, 1692 cm^−1^, and 1633 cm^−1^ due to the two carbonyl groups and (HC=CH), indicating the occurrence of an interaction between pure chitosan and phenyl acrylic acid attached to the sulfonamide group. The appearance of two bands at 1336 cm^−1^ and 1161 cm^−1^, associated with the O=S=O (symmetric and asymmetric) sulfonamide group, further supports the hypothesis that the acylation reaction between the primary amino groups of pure chitosan and the sulfonamide derivative of phenyl acrylic acid has taken place. Based on these observations, we conclude that the acylation reaction between the free primary amine groups in chitosan and the sulfonamide derivative of phenyl acrylic acid has been successfully synthesized.

#### Proton and carbon nuclear magnetic resonance studies

**Figure 2:**
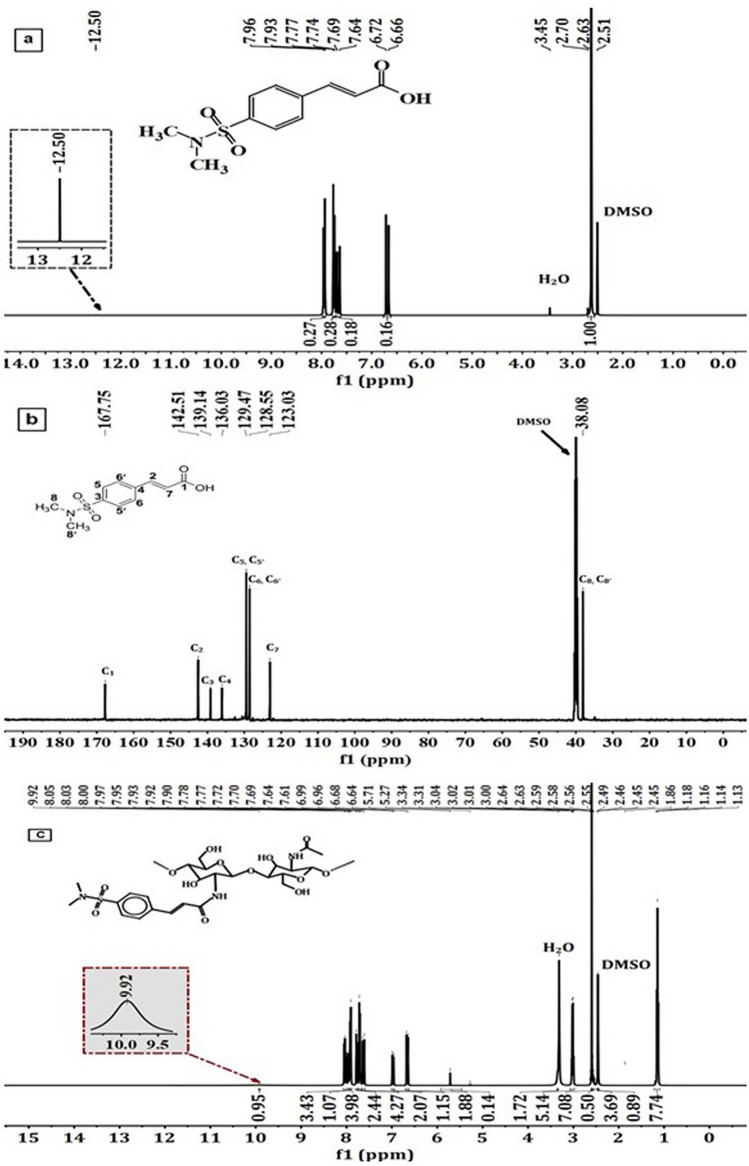
(**a**) ^1^H-NMR, (**b**) ^13^C-NMR of sulfonamide derivative (DMSA), and (**c**) ^1^H-NMR of DMS-CH derivative.

The chemical structure of the DMSA was confirmed by nuclear magnetic resonance. The ^1^H-NMR spectrum of this derivative (Fig. 2a) displayed a singlet signal at δ = 12.5 ppm related to the hydroxyl proton. Additionally, the four protons of the aromatic ring appeared as doublet-doublet at δ = 7.93 ppm and 7.77 ppm with coupling constants *J* = 8.3 Hz and *J* = 8.5 Hz, respectively. The two signals at δ = 7.64 ppm and 6.72 ppm, attributed to the two trans-olefinic protons and the aliphatic protons of dimethyl amine (–N(Me)_2_), were observed as singlet signals at δ = 2.63 ppm. To further confirm the structure of DMSA, we recorded the ^13^C-NMR spectrum (Fig. 2b), which revealed characteristic carbon signals at δ = 167.75, 142.51, 139.14, 136.03, 129.47, 129.47, 128.55, 128.55, 123.03, and 38.08 ppm. These signals were because of the carbon of the carbonyl group (C1), trans-olefinic carbons (C2, C7), aromatic carbons (C4, C3, C5, C5′, C6, C6′), and ((CH_3_)_2_N–) groups (C8, C8′), respectively. Furthermore, when comparing the ^1^H-NMR spectrum between DMS-CH (Fig. 2c) and chitosan (CH), the signals of chitosan CH^45^ were observed at δ = 1.82 ppm, representing the three protons of the N-COCH_3_ residue, and the signal at δ = 2.98 ppm was allocated to the H-2. Chemical shifts of H-3, H-4, H-5, and H-6 of the glucosamine moiety and N-acetylated glucosamine protons were represented by multiple signals ranging from δ = 3.753 ppm to δ = 4.12 ppm. Additionally, multiple signals were identified at δ = 4.4–5.4 ppm, corresponding to the H-1 protons. On the other hand, ^1^H-NMR spectrum of DMS-CH derivative did not show a singlet signal for the hydroxyl group and contained a new signal at 9.92 ppm attributed to the N–H group. Furthermore, characteristic signals were reliably detected between δ = 8.05 and 6.64 ppm, confirming the existence of the aromatic ring and the trans-olefinic protons in the newly modified polymer. The chemical shift in the aliphatic range at δ = 2.59 ppm could be because of the protons of the two groups of dimethyl (–N(CH_3_)_2_–). These findings provide evidence that native chitosan (CH) undergoes molecular interactions through an amidic bond between the free amino groups and the non-isolable intermediate acid chloride.

#### Mass spectrum

**Figure 3:**
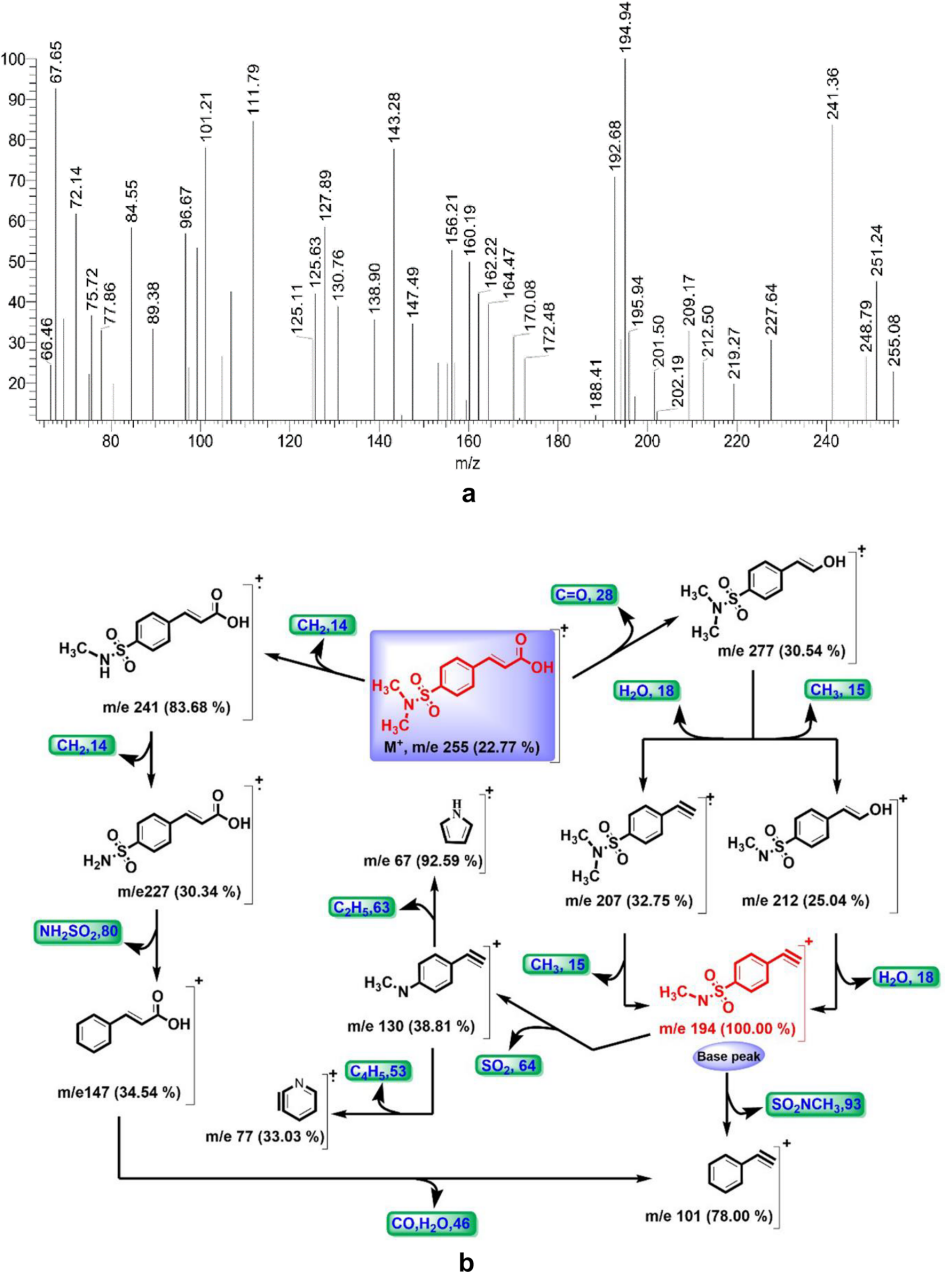
(**a**) The mass spectrum of DMSA and (**b**) Proposed fragmentation pathway of M^+^ ion derived from DMSA.

The mass spectrum of DMSA showed an ion peak at m/e 255 (22.77%) (Fig. 3a), which agrees well with the molecular formula C_11_H_13_NO_4_S proposed from elemental analysis. A base peak observed at m/e 194 (100%) was attributed to ethynylbenzene sulfonamide moiety. The fragmentation patterns (Fig. 3b) showed loss of C=O, CH_2_, CH_3_, H_2_O, NH_2_SO_2_, SO_2_, SO_2_NCH_3_, C_4_H_5_, and C_2_H_5_, where fragmentation patterns were observed at 277 (30.54%), 241 (83.68%), 212 (25.04%), 207 (32.75%), 147 (34.54%), 130 (38.81%), 101 (78.00%), 77 (33.03%), and 67 (92.59%).

#### Degree of substitution (DS) of the prepared derivative DMS-CH

In Table 1, the degree of substitution of the chitosan derivative, DMS-CH, was calculated based on the elemental C, H, and N analysis using the following equation^46^^,^^47^.

2 $$DS\% = 100 \times \left[ {\frac{{n_{1} \left( {C/N} \right)_{modified} - \left( {C/N} \right)_{unmodified} }}{{n_{2} }}} \right]$$

where (C/N)_modified_ and (C/N)_unmodified_ are the carbon over nitrogen ratio for DMS-CH and CH, respectively. n_1_ and n_2_ are the numbers of nitrogen and carbon atoms introduced to chitosan after modification, respectively.

**Table 1:** Degree of substitution of DMS-CH derivative.

Compound	Element (%)				C/N	DS(%)
	C	H	N	S		
CH	36.38	5.56	6.05	–	6.01	–
DMS-CH	48.27	4.46	4.54	12.23	10.6	42

The data in Table 1 provided additional proof for the synthesis of the DMS-CH derivative, where 12.23% sulfur was recorded by the elemental analysis of DMS-CH. This confirms the reaction between the sulfonamide derivative and chitosan. From Table 1, the DS of DMS-CH was 42%, indicating a moderate degree of substitution; consequently, free amino groups are still present in the DMS-CH derivative. This may be due to the presence of the bulky sulfonamide derivative^47^.

#### Surface morphology of DMS-CH derivative

**Figure 4:**
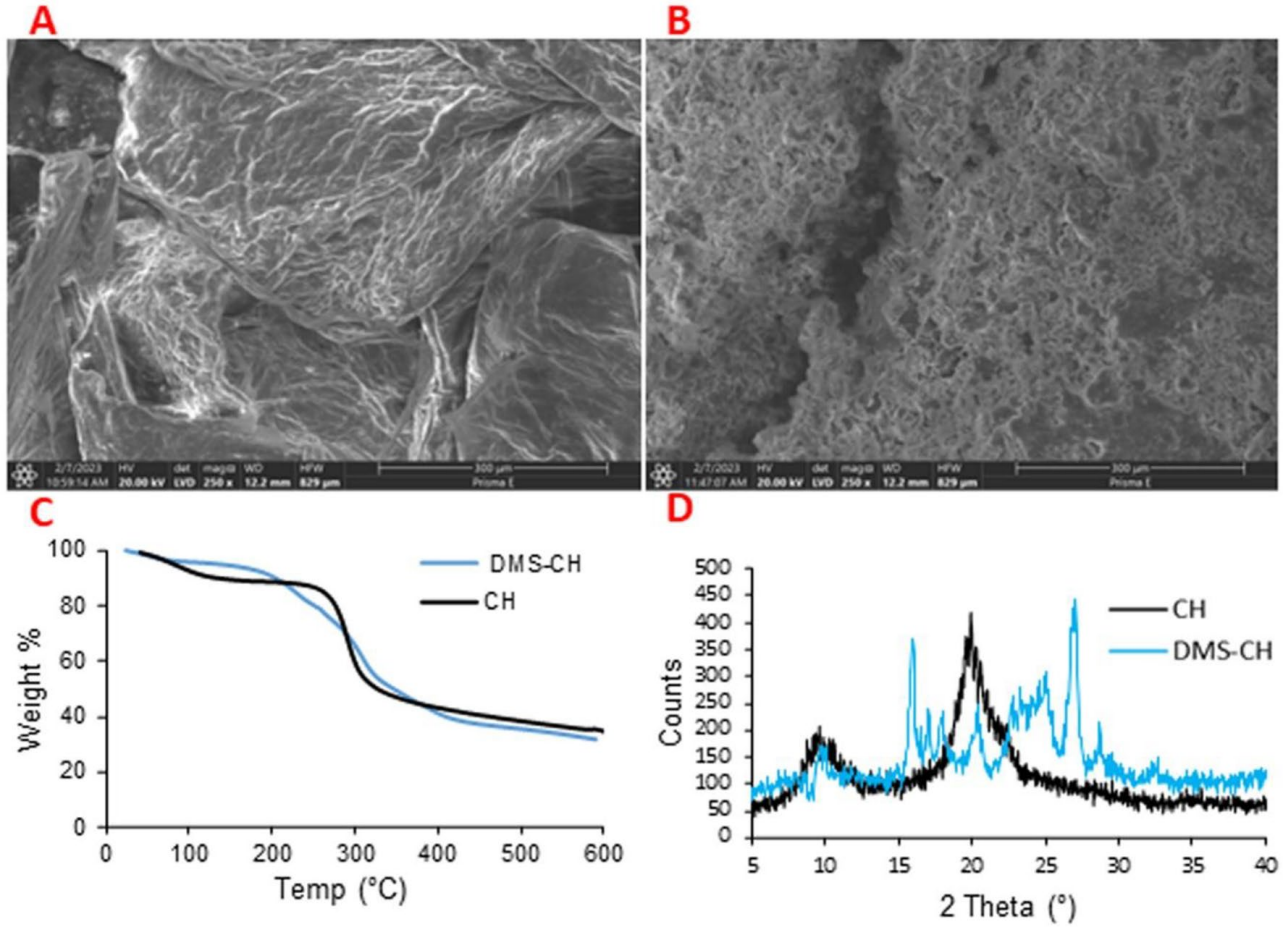
Surface morphologies for CH (**A**) and DMS-CH (**B**), TGA of CH and its sulfonamide derivative (**C**), and XRD of CH and its sulfonamide derivative (**D**).

The difference between the surface morphologies of CH and DMS-CH was investigated by high-resolution electron microscopy technique. A and B in Fig. 4 show the SEM images of CH and DMS-CH, respectively, at a magnification of 250×. The Fig. 4A indicated the smooth and nonporous surfaces of chitosan^48^. After modification with the sulfonamide derivative, a notable surface change was found. The DMS-CH surface was irregular and porous, and this was in line with the literature^49,50^.

#### Thermal analysis

One of the investigation tools that indicates the chemical modification of a certain polymer is the change in its thermal profile. As observed in Fig. 4C, the thermal profiles of the two polymers, CH and DMS-CH, exhibit differences, providing additional evidence for the synthesis of the chitosan derivative. It is evident that the initial decomposition temperature has shifted from 266 °C for chitosan, as observed in our recent works^4,51^, to 187 °C for DMS-CH. This shift demonstrates two important points: (1) the two structures of CH and DMS-CH are chemically dissimilar, and (2) the decreased thermal stability of the DMS-CH derivative compared to unmodified chitosan. At 266 °C, CH showed 15.9% weight loss, whereas DMS-CH exhibited a weight loss of 23.5%, indicating the successive release of the introduced moieties into the chitosan structure. However, no significant difference was observed in the residual weight at 600 °C for the two polymers, suggesting that they undergo similar degradation reactions at elevated temperatures. This includes the crosslinking of glucopyranose chains, leading to the formation of thermally stable network structures^52,53^. In conclusion, the introduction of the sulfonamide-based compound into the chitosan structure results in a reduction in its thermal stability, which is in line with previous findings^33^.

#### X-ray diffraction (XRD)

XRD is used to investigate the crystalline morphology of polymers. Figure 4D shows the XRD peaks for CH and DMS-CH. CH showed two peaks at 2θ = 9.6° and 20°, confirming the semicrystalline order of its structure^8,54^. The two peaks were observed in the XRD of DMS-CH in addition to new peaks of high intensity at 2θ = 16°, 25°, and 27°, indicating that DMS-CH had a higher degree of crystallinity than CH. This significant finding may be attributed to the presence of sulfonamide and amide groups in DMS-CH, and this is similar to that found in previous works^33,49^.

### Biological applications

#### Antimicrobial activity

**Figure 5:**
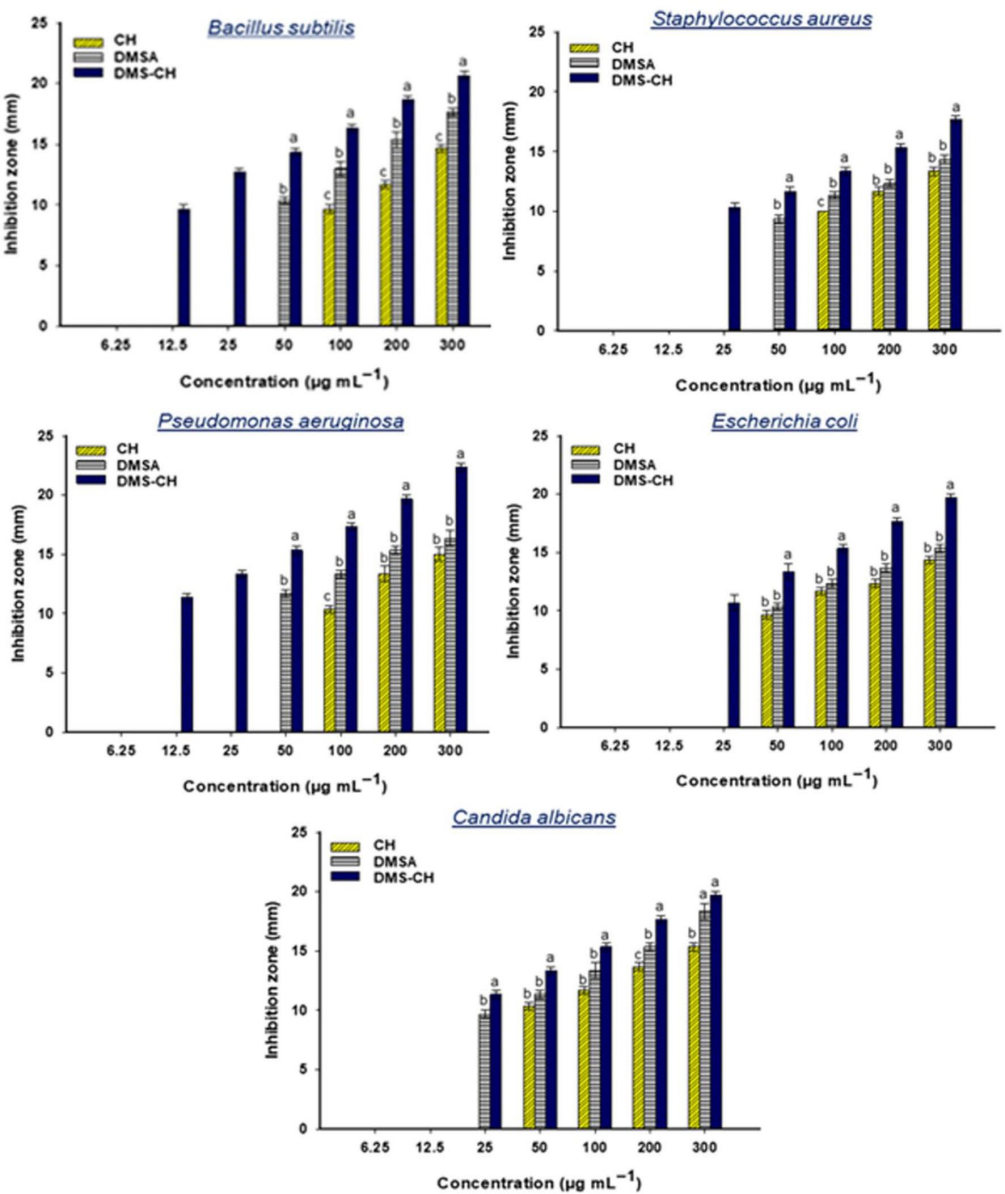
The antimicrobial activity of CH, DMSA, and DMSS-CH against different pathogenic gram-positive bacteria, *B. subtilis* and *S. aureus*, gram-negative bacteria, *P. aeruginosa* and *E. coli*, and unicellular fungi *C. albicans*. Data are statistically analyzed at *p* ≤ 0.05 using Tukey's test (n = 3, ± SD). Bars with the same letters at different concentrations mean that the data are not significantly different.

In this study, the simple agar-well diffusion test has been used to evaluate the antimicrobial properties of CH, DMSA derivative, and DMS-CH against the representative Gram-positive bacteria (*Staphylococcus aureus* and *Bacillus subtilis*), Gram-negative bacteria (*Pseudomonas aeruginosa* and *Escherichia co*li), and the fungus *Candida albicans*. Our investigations confirmed varying degrees of activity of the three tested materials against the selected microbes, where the highest activity was for DMS-CH. At the same time, pure CH showed the least activity (Tables 2, 3, Fig. 5). Recently, chemically modified chitosan derivatives have been shown to possess improved antimicrobial properties compared with the native chitosan^55^. Similarly, our modified chitosan showed enhanced antimicrobial activity; 300 µg mL^−1^ of DMS-CH increased the zones of inhibition (ZOI) by 41, 32.5, 48, 37, and 28% compared to the ZOI represented by CH against the pathogens *B. subtilis*, *S. aureus*, *P. aeruginosa*, *E. coli*, and *C. albicans*, respectively. The maximum inhibitory effect of DMS-CH was recorded at 300 µg/mL against *P. aeruginosa* (ZOI = 22.33 ± 0.57 mm)*,* while *S. aureus* showed the least sensitivity to DMS-CH (300 µg mL^−1^/ZOI = 17.66 ± 0.57 mm). Consistent with our results, 4-carboxybenzensulfonamide-chitosan was more efficient than native chitosan and presented boosted antibacterial efficiency against *S. aureus* and *E. coli* as models for Gram-positive and Gram-negative bacterial strains^56^. Besides that, the derivative DMSA showed less activity than the modified chitosan against the tested microbes. A concentration of 300 µg/mL of DMSA led to the development of ZOI: 17.6 ± 0.57, 14.3 ± 0.5, 16.3 ± 1.15, 15.33 ± 0.57 and 18.33 ± 1.15 mm versus *B. subtilis*, *S. aureus*, *P. aeruginosa*, *E. coli*, and *C. albicans*, respectively.

**Table 2:** Antibacterial activity of synthesized materials (CH, DMSA, and DMSCH) against Gram-positive strains, *B. subtilis* and *S. aureus.*

Conc (µg/mL)	*B. subtilis*			*S. aureus*		
	CH	DMSA	DMS-CH	CH	DMSA	DMS-CH
300	14.7 ± 0.6	17.7 ± 0.6	20.7 ± 0.6	13.3 ± 0.6	14.3 ± 0.6	17.7 ± 0.6
200	11.7 ± 0.6	15.3 ± 1.2	18.7 ± 0.6	11.7 ± 0.6	12.3 ± 0.6	15.3 ± 0.6
100	9.6 ± 0.6	13.0 ± 1.0	16.3 ± 0.6	0.0 ± 0.0	11.3 ± 0.6	13.3 ± 0.6
50	0.0 ± 0.0	10.3 ± 0.6	14.3 ± 0.6	0.0 ± 0.0	9.3 ± 0.6	11.7 ± 0.6
25	0.0 ± 0.0	0.0 ± 0.0	12.7 ± 0.6	0.0 ± 0.0	0.0 ± 0.0	10.3 ± 0.6
12.5	0.0 ± 0.0	0.0 ± 0.0	9.7 ± 0.6	0.0 ± 0.0	0.0 ± 0.0	0.0 ± 0.0
6.25	0.0 ± 0.0	0.0 ± 0.0	0.0 ± 0.0	0.0 ± 0.0	0.0 ± 0.0	0.0 ± 0.0

Data are represented by the means of three replicates ± SD.

**Table 3:** Antimicrobial activity of synthesized materials (CH, DMSA, and DMSCH) against Gram-negative bacteria (*P*. *aeruginosa* and *E. coli*) and unicellular fungi (*C. albicans).*

Conc (µg/mL)	*P*. *aeruginosa*			*E. coli*			*C. albicans*		
	CH	DMSA	DMS-CH	CH	DMSA	DMS-CH	CH	DMSA	DMS-CH
300	15.0 ± 1.0	16.3 ± 1.2	22.3 ± 0.6	14.3 ± 0.6	15.3 ± 0.6	19.7 ± 0.3	15.3 ± 0.6	18.3 ± 1.2	19.7 ± 0.6
200	13.3 ± 1.2	15.3 ± 0.6	19.7 ± 0.6	12.3 ± 0.6	13.7 ± 0.6	17.7 ± 0.3	13.7 ± 0.6	15.3 ± 0.6	17.7 ± 0.6
100	10.3 ± 0.6	13.3 ± 0.6	17.3 ± 0.6	11.7 ± 0.6	12.3 ± 0.6	15.3 ± 0.3	11.7 ± 0.6	13.3 ± 1.2	15.3 ± 0.6
50	0.0 ± 0.0	11.7 ± 0.6	15.3 ± 0.6	9.7 ± 0.6	10.3 ± 0.6	13.3 ± 0.7	10.3 ± 0.6	11.3 ± 0.6	13.3 ± 0.6
25	0.0 ± 0.0	0.0 ± 0.0	13.3 ± 0.6	0.0 ± 0.0	0.0 ± 0.0	10.7 ± 0.7	0.0 ± 0.0	9.7 ± 0.6	11.3 ± 0.6
12.5	0.0 ± 0.0	0.0 ± 0.0	11.3 ± 0.6	0.0 ± 0.0	0.0 ± 0.0	0.0 ± 0.0	0.0 ± 0.0	0.0 ± 0.0	0.0 ± 0.0
6.25	0.0 ± 0.0	0.0 ± 0.0	0.0 ± 0.0	0.0 ± 0.0	0.0 ± 0.0	0.0 ± 0.0	0.0 ± 0.0	0.0 ± 0.0	0.0 ± 0.0

Data are represented by the means of three replicates ± SD.

Although pure chitosan exhibited lower antimicrobial activity compared to the DMSA derivative and modified chitosan, a concentration of 300 µg/mL of native chitosan showed considerable activity against *B. subtilis*, *S. aureus*, *P. aeruginosa*, *E. coli*, and *C. albicans,* recording ZOIs values of 14. 6 ± 0.57, 13.3 ± 0.57, 15 ± 1.0, 14.33 ± 0.57, and 15.33 ± 0.5 mm, respectively (Fig. 5). Given the broad-spectrum activity of CH, DMSA, and DMS-CH against various clinical pathogens, it is necessary to assign their minimum inhibitory concentration (MIC) values to propose and study their therapeutic use^57^. The MIC value for DMS-CH was as low as 12.5 µg/mL against *B. subtilis* and *P*. *aeruginosa*, while it increased to 25 µg/mL against *S. aureus*, *E. coli*, and *C. albicans*. The derivative DMSA displayed a MIC value of 50 µg mL^−1^ for all tested strains, except for *C. albicans*, which was 25 µg/mL compared to the DMSA and DMS-CH, native chitosan exhibited the weakest activity against the tested microbes, while it had the highest MIC value of 100 µg/mL for all tested microbes, except for *E. coli* and *C. albicans*, where it was 50 µg/mL_._ Likewise, the recently prepared chitosan-benzene-sulfonamide derivative manifested enhanced antibacterial activity against *S. aureus* and *E. coli* with a MIC value of 50 µg/mL^58^. Notably, Gram-negative bacteria were more sensitive to our preparations than Gram-negative strains. This difference may be attributed to the difference in the composition of cell walls, as Gram-negative cell walls have a thinner peptidoglycan layer and contain lipopolysaccharides, while Gram-positive bacterial cell walls have a thicker peptidoglycan layer^59^. Additionally, chitosan derivatives could coat cell surfaces with a thick polymer film, covering porins in the outer membrane of Gram-negative bacteria and inhibiting the exchange of nutrients, ultimately leading to cell death^60^. Moreover, the positively charged chitosan interacts electrostatically with the negatively charged surface of bacteria, disrupting the cytoplasmic membrane, facilitating the infiltration of cellular components, and allowing chitosan to enter the bacterial cell^55^. Interestingly, chitosan offers significant anti-biofilm characteristics resulting from electrostatic interactions between the negatively charged components of biofilm (e-DNA, ESP, and extracellular proteins) and the positively charged chitosan^61,62^.

#### Cytotoxicity

**Figure 6:**
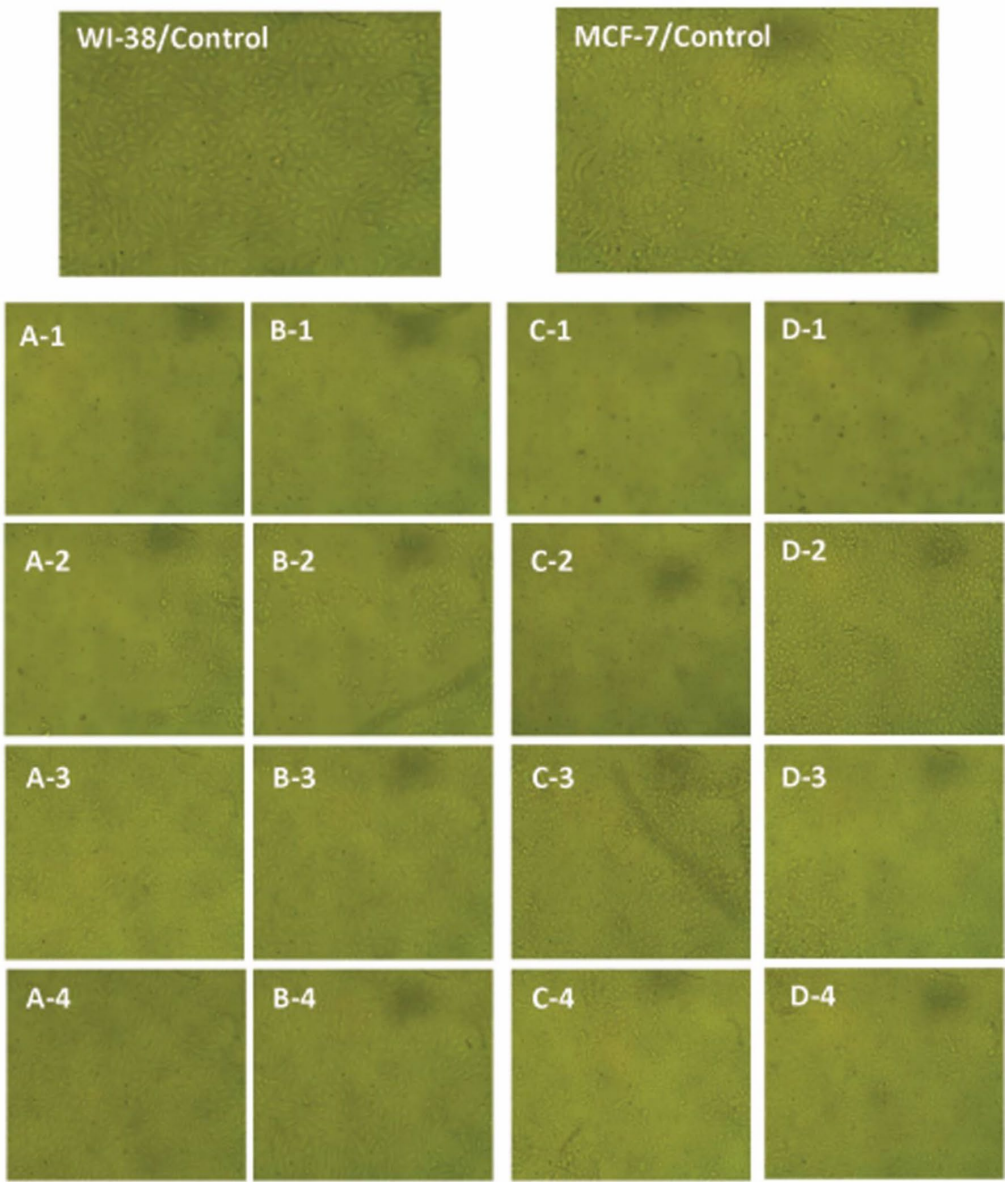
Morphological changes of cell lines WI-38 and MCF-7 after treatment with different concentrations of DMSA and DMS-CH. 1, 2, 3, and 4 represented the concentrations of 1000, 250, 62.5, and 31.25 µg/mL respectively. A and B are the effect of DMSA and DMS-CH on the morphology of normal cell lines Wi38, whereas C and D are the effect of DMSA and DMS-CH on the morphology of cancer cell lines MCF-7.

**Figure 7:**
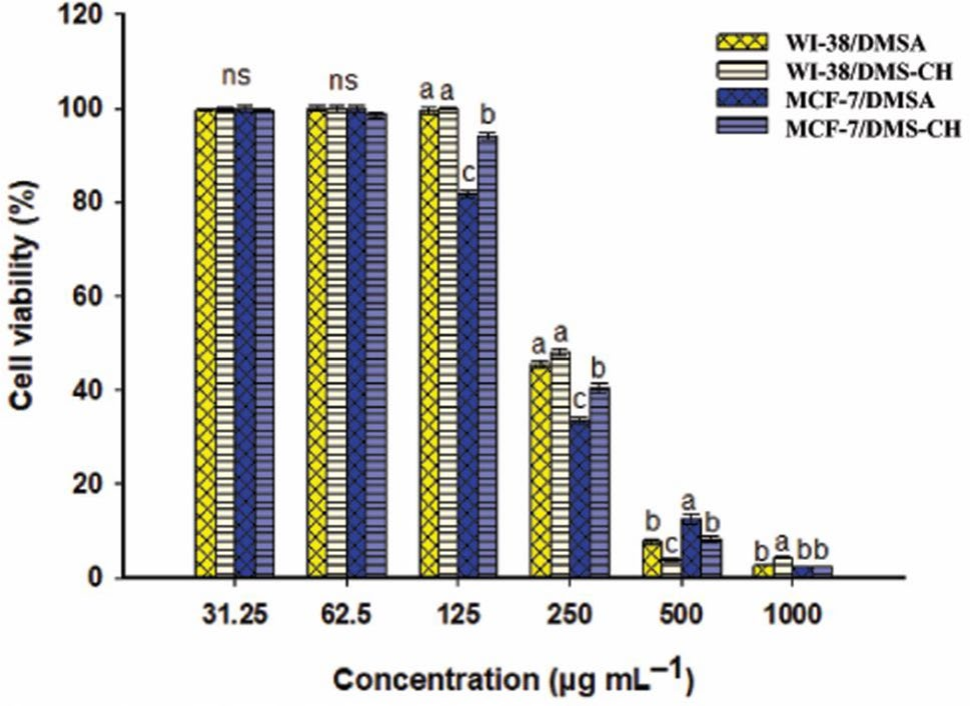
The cell viability assay using the MTT method of cancerous cell lines (MCF-7) and normal fibroblast cells (WI-38) due to treatment with different concentrations of DMSA and DMS-CH.

Colorimetric readouts of cellular activity are one of the most important methods used in cytotoxicity studies. The MTT test, which is the most widely used, has been efficiently used to evaluate the biocompatibility of chitosan and its derivatives^63^. The current investigations were based on the MTT assay to quantify the anti-proliferative potential of the newly modified chitosan (DMS-CH), the DMSA derivative, and pure chitosan (CH) against breast adenocarcinoma cell line “MCF-7” and normal lung fibroblast cell line “WI-38”. The prepared concentrations of all tested substances, ranging from 31.25 to 1000 μg/mL, showed an inhibitory effect on the vitality of cancer cells in a concentration-dependent way (Table 4). Microscopic examination confirmed the deformation and loss of the distinctive monolayer of malignant cells, which also appeared granular and shrunken (Fig. 6). In comparison, the normal fibroblasts “WI-38” displayed less sensitivity to the tested substances. Their phenotypic traits began to disfigure, and their viability declined only at higher concentrations compared to the corresponding concentrations required to block the proliferation of “MCF-7” adenocarcinoma cells. The IC50 “the concentration needed for inhibiting half of the population” is traditionally used to define the efficacy of chemicals/drugs based on cytotoxicity assays^64^. Regarding the cancerous cell line “MCF-7”, our measurements revealed the IC50 values 209.7 ± 5.2, 193.49 ± 4.54, and 225.47 ± 2.51 µg/mL assigned for CH, DMSA, and DMS-CH, respectively. In contrast, the IC50 amounts for the normal cells “WI-38” increased to 323.35 ± 9.12, 238.62 ± 2.31, and 247.205 ± 27.54 µg/mL corresponding to CH, DMSA, and DMS-CH, respectively (Fig. 7). The IC50 value and toxicological properties of pure chitosan against “MCF-7” and “WI-38” cells were evaluated in our recent work^51^. Our findings disclosed that the IC50 measurements of "WI-38" normal clones increased by 54.19%, 23.32%, and 9.63% compared to the corresponding values for the “MCF-7” malignant cell lines treated with CH, DMSA, and DMS-CH, respectively. This critical difference can be exploited to suggest the possibility of using these substances as chemotherapeutic agents in safe doses^41^. Compatible with our results, Omer et al. developed chitosan coupled with benzene-sulfonamide derivative (CH-SB) and examined the cytotoxic properties of chitosan (CH) and CH-SB against HepG-2 (hepatocellular carcinoma) and HSF (normal human skin fibroblasts) cell lines. They reported the anti-proliferative potency of CH-SB against HepG-2 cells (IC50 = 109.6 µg/mL) and mild toxicity against normal HSF cells (IC50 = 726.7 µg/mL), while CH displayed modest antitumor properties against normal somatic skin cells (HSF) and hepatoma (HepG-2) cell lines recording IC50 values of 875.21 and 755.9 µg/mL, respectively^58^. In the same regard, newly formulated chitosan-β-ketosulfone derivatives in little concentrations manifested significant antitumor potential against the three mammalian cancer cell lines breast carcinoma (MCF-7), liver hepatocellular carcinoma (HEPG2), and colon carcinoma (HCT)^65^. Modified chitosan selectively penetrates the cancer cell through the plasma membrane, interfering with the flow of genetic information from DNA to RNA for protein synthesis and obstructing hormone synthesis. This disruption of normal cellular processes suppresses the growth of cancer cells^66^. Moreover, the positive charge of modified chitosan resulting from the amino group could facilitate its penetration into the negatively charged plasma membranes of cancer cells^67^. Malignant cells having more negative charges compared to their normal cell counterparts, which make them a selective target for positively charged modified chitosan, which interferes with metabolic and growth processes and induces apoptosis in cancer cells. Additionally, chitosan can improve the immune response, boost the activity of phagocytic cells, and prevent the spread and infiltration of cancer into adjacent tissues by disrupting the formation of vascular endothelial cells^34,68^.

**Table 4:** The cell viability of normal cell lines (WI-38) and cancer cell lines (MCF-7) due to treatment with different concentrations of DMSA and DMS-CH.

Conc	WI-38		MCF-7	
	DMSA	DMS-CH	DMSA	DMS-CH
1000	2.6 ± 0.1	4.3 ± 0.4	2.3 ± 0.1	2.4 ± 0.2
500	7.7 ± 1.1	3.8 ± 0.8	12.5 ± 1.7	8.2 ± 1.3
250	45.6 ± 1.2	48.1 ± 1.3	33.6 ± 1.4	40.4 ± 1.6
125	99.6 ± 1.2	99.9 ± 0.4	81.7 ± 0.9	94.1 ± 1.5
62.5	99.9 ± 0.9	99.9 ± 1.2	99.9 ± 1.2	98.8 ± 0.9
31.25	99.7 ± 0.5	99.9 ± 0.9	99.9 ± 1.1	99.5 ± 0.6

## Conclusions

In this work, we successfully designed a new biopolymer bearing a sulfonamide group, which has been reported as a sulfa drug. The new biopolymer derivative was synthesized by a simple reaction between chitosan and N,N-dimethyl sulfonyl phenyl acryloyl chloride. The structure of the new derivative was confirmed through FT-IR, elemental analysis, and ^1^H-NMR. The results of this study revealed that the degree of substitution of DMSA-modified chitosan (DMS-CH) was 42%. The surface of DMS-CH exhibited irregularities and porosity, indicating structural changes induced by the introduction of the sulfonamide-based compound into the chitosan (CH) framework. Furthermore, the thermal stability of the modified chitosan was reduced compared to pure chitosan, suggesting alterations in its chemical properties. Interestingly, DMS-CH displayed a higher degree of crystallinity than CH, implying changes in its physical characteristics. In addition, the modification of chitosan with sulfonamides moiety synergizes its effectiveness against a wide group of selected clinical microbes, including Gram-negative bacteria such as *Pseudomonas aeruginosa* and *Escherichia coli*, Gram-positive bacteria like *Bacillus subtilis* and *Staphylococcus aureus*, and the dimorphic fungus *Candida albicans* (MIC = 12.5–25 µg/mL). In contrast, native chitosan showed lower activity against the tested strains (MIC = 50–100 µg/mL). Furthermore, compared to pure chitosan, the modified chitosan displayed improved selective toxicity against the breast adenocarcinoma cell lines (MCF-7) with an IC50 value of 225.47 µg/mL, while maintaining safety limits against normal lung fibroblasts (WI-38). These findings suggest the potential use of the newly modified chitosan as a versatile material in biomedical settings, however, addressing limitations related to the mechanical properties and long-term stability of the DMS-CH derivative, as well as conducting comprehensive preclinical cytotoxicity studies including in vivo models, will be our primary focus in future research.

## Data Availability

Request for the raw data used in this study will be fulfilled from the corresponding author (Ahmed G. Ibrahim).

## References

[1] Yang Z (2014). Evaluation of a novel chitosan-based flocculant with high flocculation performance, low toxicity and good floc properties. J. Hazard. Mater..

[2] Poonguzhali R, Basha SK, Kumari VS (2017). Synthesis and characterization of chitosan-PVP-nanocellulose composites for in-vitro wound dressing application. Int. J. Biol. Macromol..

[3] Khan MUA (2024). Fundamental properties of smart hydrogels for tissue engineering applications: A review. Int. J. Biol. Macromol..

[4] Mohamed AE (2022). Synthesis and characterization of new functionalized chitosan and its antimicrobial and in-vitro release behavior from topical gel. Int. J. Biol. Macromol..

[5] Khan MUA (2023). Recent perspective of polymeric biomaterial in tissue engineering—A review. Mater. Today Chem..

[6] Ibrahim AG, Saleh AS, Elsharma EM, Metwally E, Siyam T (2019). Chitosan-g-maleic acid for effective removal of copper and nickel ions from their solutions. Int. J. Biol. Macromol..

[7] Dragostin OM (2015). Development and characterization of novel films based on sulfonamide-chitosan derivatives for potential wound dressing. Int. J. Mol. Sci..

[8] Ibrahim AG (2023). Development of a chitosan derivative bearing the thiadiazole moiety and evaluation of its antifungal and larvicidal efficacy. Polym. Bull..

[9] Khan R (2023). Fabrication of amine-functionalized and multi-layered PAN-(TiO(2))-gelatin nanofibrous wound dressing: In-vitro evaluation. Int. J. Biol. Macromol..

[10] Pestov AV, Ezhikova MA, Kodess MI, Azarova YA, Bratskaya SY (2014). Preparation of a sorbent for metal ions based on N-(5-methylimidazol-4-ylmethyl) chitosan with medium degree of substitution. Russ. J. Appl. Chem..

[11] Přichystalová H (2014). Synthesis, characterization and antibacterial activity of new fluorescent chitosan derivatives. Int. J. Biol. Macromol..

[12] Ahmed ME, Mohamed HM, Mohamed MI, Kandile NG (2020). Sustainable antimicrobial modified chitosan and its nanoparticles hydrogels: Synthesis and characterization. Int. J. Biol. Macromol..

[13] Cui J, Sun Y, Wang L, Tan W, Guo Z (2023). Preparation of chitosan derivatives containing aromatic five-membered heterocycles for efficient antimicrobial and antioxidant activities. Int. J. Biol. Macromol..

[14] Lee D-S, Woo J-Y, Ahn C-B, Je J-Y (2014). Chitosan–hydroxycinnamic acid conjugates: Preparation, antioxidant and antimicrobial activity. Food Chem..

[15] Yue L (2021). Preparation and characterization of chitosan oligosaccharide derivatives containing cinnamyl moieties with enhanced antibacterial activities. Lwt.

[16] Wei L (2017). Synthesis, characterization, and the antioxidant activity of double quaternized chitosan derivatives. Molecules.

[17] Vadivel T, Dhamodaran M (2016). Synthesis, characterization and antibacterial studies of ruthenium (III) complexes derived from chitosan Schiff base. Int. J. Biol. Macromol..

[18] Gutha Y, Munagapati VS (2016). Removal of Pb (II) ions by using magnetic chitosan-4-((pyridin-2-ylimino) methyl) benzaldehyde Schiff’s base. Int. J. Biol. Macromol..

[19] Shahraki S, Delarami HS, Khosravi F (2019). Synthesis and characterization of an adsorptive Schiff base-chitosan nanocomposite for removal of Pb (II) ion from aqueous media. Int. J. Biol. Macromol..

[20] de Araújo EL, Barbosa HFG, Dockal ER, Cavalheiro ÉTG (2017). Synthesis, characterization and biological activity of Cu (II), Ni (II) and Zn (II) complexes of biopolymeric Schiff bases of salicylaldehydes and chitosan. Int. J. Biol. Macromol..

[21] Zidan TA, Abdelhamid AE, Zaki EG (2020). N-Aminorhodanine modified chitosan hydrogel for antibacterial and copper ions removal from aqueous solutions. Int. J. Biol. Macromol..

[22] Hamza MF (2022). Functionalized biobased composite for metal decontamination—Insight on uranium and application to water samples collected from wells in mining areas (Sinai, Egypt). Chem. Eng. J..

[23] Hamza MF (2022). Functionalization of magnetic chitosan microparticles for high-performance removal of chromate from aqueous solutions and tannery effluent. Chem. Eng. J..

[24] Frost CG, Hartley JP, Griffin D (2002). Catalytic arylation of sulfamoyl chlorides: A practical synthesis of sulfonamides. Synlett.

[25] Zani F, Vicini P (1998). Antimicrobial activity of some 1, 2-benzisothiazoles having a benzenesulfonamide moiety. Arch. Pharm.: Int. J. Pharm. Med. Chem..

[26] Maren TH (1976). Relations between structure and biological activity of sulfonamides. Annu. Rev. Pharmacol. Toxicol..

[27] Renzi G, Scozzafava A, Supuran CT (2000). Carbonic anhydrase inhibitors: topical sulfonamide antiglaucoma agents incorporating secondary amine moieties. Bioorganic Med. Chem. Lett..

[28] Penning TD (1997). Synthesis and biological evaluation of the 1, 5-diarylpyrazole class of cyclooxygenase-2 inhibitors: Identification of 4-[5-(4-methylphenyl)-3-(trifluoromethyl)-1 H-pyrazol-1-yl] benzenesulfonamide (SC-58635, celecoxib). J. Med. Chem..

[29] Yoshino H (1992). Novel sulfonamides as potential, systemically active antitumor agents. J. Med. Chem..

[30] Doungsoongnuen S (2011). Investigation on biological activities of anthranilic acid sulfonamide analogs. EXCLI J..

[31] El-Sayed NS, El-Bendary ER, El-Ashry SM, El-Kerdawy MM (2011). Synthesis and antitumor activity of new sulfonamide derivatives of thiadiazolo [3, 2-a] pyrimidines. Eur. J. Med. Chem..

[32] Roush WR (1998). Vinyl sulfonate esters and vinyl sulfonamides: Potent, irreversible inhibitors of cysteine proteases [10]. J. Am. Chem. Soc..

[33] Kandile NG (2023). New sustainable antimicrobial chitosan hydrogels based on sulfonamides and its nanocomposites: Fabrication and characterization. Int. J. Biol. Macromol..

[34] Ibrahim AG (2022). New thiadiazole modified chitosan derivative to control the growth of human pathogenic microbes and cancer cell lines. Sci. Rep..

[35] Wang W, Bo S, Li S, Qin W (1991). Determination of the Mark-Houwink equation for chitosans with different degrees of deacetylation. Int. J. Biol. Macromol..

[36] Mi Y (2020). New synthetic chitosan derivatives bearing benzenoid/heterocyclic moieties with enhanced antioxidant and antifungal activities. Carbohydr. Polym..

[37] Abdo AM (2021). Green synthesis of zinc oxide nanoparticles (ZnO-NPs) by pseudomonas aeruginosa and their activity against pathogenic microbes and common house mosquito, *Culex pipiens*. Materials.

[38] Hamza MF (2022). U(VI) and Th(IV) recovery using silica beads functionalized with urea- or thiourea-based polymers—Application to ore leachate. Sci. Total Environ..

[39] Ibrahim AG (2023). New functionalized chitosan with thio-thiadiazole derivative with enhanced inhibition of pathogenic bacteria, plant threatening fungi, and improvement of seed germination. Chemistry.

[40] Fouda A (2022). Endophytic bacterial strain, Brevibacillus brevis-mediated green synthesis of copper oxide nanoparticles, characterization, antifungal, in vitro cytotoxicity, and larvicidal activity. Green Process. Synth..

[41] Fouda A (2022). Green synthesis of gold nanoparticles by aqueous extract of *Zingiber officinale*: characterization and insight into antimicrobial, antioxidant, and in vitro cytotoxic activities. Appl. Sci..

[42] Nassar A-RA, Eid AM, Atta HM, El Naghy WS, Fouda A (2023). Exploring the antimicrobial, antioxidant, anticancer, biocompatibility, and larvicidal activities of selenium nanoparticles fabricated by endophytic fungal strain *Penicillium verhagenii*. Sci. Rep..

[43] Halawa AH (2019). Rational design and synthesis of diverse pyrimidine molecules bearing sulfonamide moiety as novel ERK inhibitors. Int. J. Mol. Sci..

[44] Halawa AH (2020). Synthesis, anticancer evaluation and molecular docking studies of new heterocycles linked to sulfonamide moiety as novel human topoisomerase types I and II poisons. Bioorganic Chem..

[45] Yang F (2023). A novel water-soluble chitosan grafted with nerol: Synthesis, characterization and biological activity. Int. J. Biol. Macromol..

[46] Baran T, Menteş A (2015). Cu(II) and Pd(II) complexes of water soluble O-carboxymethyl chitosan Schiff bases: Synthesis, characterization. Int. J. Biol. Macromol..

[47] de Araújo Braz EM (2018). Modified chitosan-based bioactive material for antimicrobial application: Synthesis and characterization. Int. J. Biol. Macromol..

[48] Li K, Zhu J, Guan G, Wu H (2019). Preparation of chitosan-sodium alginate films through layer-by-layer assembly and ferulic acid crosslinking: Film properties, characterization, and formation mechanism. Int. J. Biol. Macromol..

[49] Elzamly RA (2021). New sustainable chemically modified chitosan derivatives for different applications: Synthesis and characterization. Arab. J. Chem..

[50] Chen L, Tang J, Wu S, Wang S, Ren Z (2022). Selective removal of Au (III) from wastewater by pyridine-modified chitosan. Carbohydr. Polym..

[51] Hamodin AG, Elgammal WE, Eid AM, Ibrahim AG (2023). Synthesis, characterization, and biological evaluation of new chitosan derivative bearing diphenyl pyrazole moiety. Int. J. Biol. Macromol..

[52] Zawadzki J, Kaczmarek H (2010). Thermal treatment of chitosan in various conditions. Carbohydr. Polym..

[53] López FA, Mercê ALR, Alguacil FJ, López-Delgado A (2008). A kinetic study on the thermal behaviour of chitosan. J. Therm. Anal. Calorim..

[54] Kumar S, Koh J (2012). Physiochemical, optical and biological activity of chitosan-chromone derivative for biomedical applications. Int. J. Mol. Sci..

[55] Yan D (2021). Antimicrobial properties of chitosan and chitosan derivatives in the treatment of enteric infections. Molecules.

[56] Suvannasara P, Juntapram K, Praphairaksit N, Siralertmukul K, Muangsin N (2013). Mucoadhesive 4-carboxybenzenesulfonamide-chitosan with antibacterial properties. Carbohydr. Polym..

[57] Saied E (2021). The catalytic activity of biosynthesized magnesium oxide nanoparticles (MgO-NPs) for inhibiting the growth of pathogenic microbes, tanning effluent treatment, and chromium ion removal. Catalysts.

[58] Omer AM (2023). Novel cytocompatible chitosan Schiff base derivative as a potent antibacterial, antidiabetic, and anticancer agent. Arab. J. Sci. Eng..

[59] Hamza MF (2021). Phosphorylation of guar gum/magnetite/chitosan nanocomposites for uranium (VI) sorption and antibacterial applications. Molecules.

[60] Feng P (2021). Chitosan-based functional materials for skin wound repair: Mechanisms and applications. Front. Bioeng. Biotechnol..

[61] Jamil B (2016). Development of cefotaxime impregnated chitosan as nano-antibiotics: De novo strategy to combat biofilm forming multi-drug resistant pathogens. Front. Microbiol..

[62] Khan MUA (2021). Smart and pH-sensitive rGO/Arabinoxylan/chitosan composite for wound dressing: In-vitro drug delivery, antibacterial activity, and biological activities. Int. J. Biol. Macromol..

[63] Frigaard J, Jensen JL, Galtung HK, Hiorth M (2022). The potential of chitosan in nanomedicine: An overview of the cytotoxicity of chitosan based nanoparticles. Front. Pharmacol..

[64] Larsson P (2020). Optimization of cell viability assays to improve replicability and reproducibility of cancer drug sensitivity screens. Sci. Rep..

[65] Alamry KA (2018). Potential anti-cancer performance of chitosan-based β-ketosulfone derivatives. Cogent Chem..

[66] Adhikari HS, Yadav PN (2018). Anticancer activity of chitosan, chitosan derivatives, and their mechanism of action. Int. J. Biomater..

[67] Zubareva A, Shagdarova B, Varlamov V, Kashirina E, Svirshchevskaya E (2017). Penetration and toxicity of chitosan and its derivatives. Eur. Polym. J..

[68] Chen Q (2022). Progress in research of chitosan chemical modification technologies and their applications. Mar. Drugs.

